# Psychedelic-assisted psychotherapy: hope and dilemma

**DOI:** 10.1016/j.lanepe.2023.100727

**Published:** 2023-09-04

**Authors:** The Lancet Regional Health – Europe

Psychedelic-assisted psychotherapy is an emerging therapeutic approach that combines the use of psychedelics (a subclass of hallucinogenic drugs) and psychotherapy for the treatment of post-traumatic stress disorder (PTSD) and treatment-resistant depression. Compared with current treatments for depression, psychedelic-assisted psychotherapy has a more direct impact on brain activity, which can result in profound insights and promote introspection, emotional release, and cognitive shifts. Most importantly, it requires fewer treatment regimens, often just one, and in a yet-to-be-published study, the therapeutic benefits have been shown to result in sustained remission of depression at 18 months following a single treatment.

The early, promising effect of single-dose psilocybin for treatment-resistant depression in clinical trials has provided new hope for the treatment for mental health conditions. Australia has become the first country to approve the prescribing of two psychedelic substances, 3,4-methylenedioxymethamphetamine (MDMA; colloquially known as ecstasy or molly) and psilocybin (a compound derived from magic mushrooms), for the treatment of PTSD and treatment-resistant depression. It is optimistically anticipated that the US Food and Drug Administration (FDA) will also grant approval by 2024. In contrast, the European Medicines Agency (EMA) is proceeding with caution and has outlined a number of concerns around the approval of psychedelics for medical use in the European Union (EU).

The discourse surrounding the application of psychedelic-assisted psychotherapy for clinical use has created a dilemma and sparked debate among mental health professionals, researchers, and patrons of psychedelics, effectively diving them into two factions: those who endorse this innovative treatment and those who are sceptical. Both sides are supported by equally valid arguments. On one hand, proponents emphasize the long-lasting positive effect of the treatment and meaningful progress after just one treatment. On the other hand, critics draw attention to the paucity of substantial evidence due to small sample sizes in trials, the absence of clarity regarding ideal candidates for this treatment, and potential for adverse experiences in some patients, leading to exacerbated psychological challenges, commonly referred to as so-called ‘bad trips’.

Another primary concern within the medical community is that the treatment protocol is complex. Due to the psychotherapy element involved, a rigid set of guidelines cannot be easily established. Instead, therapy needs to be tailored to each individual, which places greater accountability on medical professionals with greater time demands. Additionally, there are logistical and training challenges associated with the integration of this novel treatment approach into existing health-care systems. Training therapists and health-care professionals to administer psychedelic-assisted therapy would require the provision of specialised education, which is not readily available.

More evidence is needed, with larger trials and observational studies on the follow-up of the impact of this treatment. However, questions remain as to whether it would be justified to overlook the potential of psychedelic-assisted psychotherapy for the treatment of mental health conditions—especially when, at present, treatment options for PTSD and treatment-resistant depression are far from ideal, and relapse is common. More than 30 drugs that target depression have been approved by the FDA; however, an analysis of 21 antidepressants found their efficacy in adults with major depressive disorder, although better than placebo, tended to be modest with various side-effects.

There is an urgent unmet need to develop and test more treatment options as almost one billion people have mental health conditions globally, and in the EU, mental health problems affect more than one in six people, incurring an economic cost that exceeds 4% of gross domestic product.

It is estimated that the psychedelics market could be worth more than US$8 billion by 2028 and currently 127 clinical trials for psilocybin and 88 for MDMA are listed on ClinicalTrials.gov, not only for PTSD and depression, but also for various other conditions, including alcohol use disorders, addiction, social anxiety, and enhancement of spirituality. After a research hiatus due to legal, political, and societal factors in the 1960s and 1970s, renewed interest in psychedelics promises to expedite our understanding of their mechanism of action and their applications across various clinical contexts. This resurgence could potentially lead to significant breakthrough treatments for mental health conditions while also contributing to the advancement of spiritual wellbeing.

The long-term effectiveness of psychedelic-assisted psychotherapy and its potential to outperform existing medications for PTSD and treatment-resistant depression can only be determined through more trials and subsequent observational studies. The resolution of this dilemma thus hinges on more evidence and it will take time to accumulate adequate data. Numerous uncertainties remain, and decisions about approval for medical usage are complex. In the interim, it is crucial to exercise caution in establishing practical treatment guidelines and in training health-care professionals to administer this treatment.

